# Associations of modern initial antiretroviral drug regimens with all-cause mortality in adults with HIV in Europe and North America: a cohort study

**DOI:** 10.1016/S2352-3018(22)00046-7

**Published:** 2022-05-31

**Authors:** Adam Trickey, Lei Zhang, M John Gill, Fabrice Bonnet, Greer Burkholder, Antonella Castagna, Matthias Cavassini, Piotr Cichon, Heidi Crane, Pere Domingo, Sophie Grabar, Jodie Guest, Niels Obel, Mina Psichogiou, Marta Rava, Peter Reiss, Christopher T Rentsch, Melchor Riera, Gundolf Schuettfort, Michael J Silverberg, Colette Smith, Melanie Stecher, Timothy R Sterling, Suzanne M Ingle, Caroline A Sabin, Jonathan A C Sterne

**Affiliations:** a Population Health Sciences, University of Bristol, Bristol, UK; b Department of Medicine, University of Calgary, South Alberta HIV Clinic, Calgary, AB, Canada; c University of Bordeaux, Institut de santé publique, d’épidémiologie et de développement, Institut National de la Santé et de la Recherche Médicale (INSERM) U1219, Bordeaux, France; d Centre Hospitalier Universitaire de Bordeaux, Bordeaux, France; e Division of Infectious Diseases, University of Alabama at Birmingham, Birmingham, AL, USA; f Institute of Infectious Diseases, University vita E Salute, Milan, Italy; g Division of Infectious Diseases, Lausanne University Hospital, Lausanne, Switzerland; h Infectious Diseases Outpatient Clinic, Otto-Wagner Hospital, Vienna, Austria; i Division of Infectious Diseases, Department of Medicine University of Washington, Seattle, WA, USA; j Department of Infectious Diseases, Santa Creu i Sant Pau Hospital, Barcelona, Spain; k Sorbonne Université, INSERM, Institut Pierre Louis d'Épidémiologie et de Santé Publique, Paris, France; l Department of Public Health, AP-HP, St Antoine Hospital, Paris, France; m Atlanta Veterans Association Medical Center, Decatur, GA, USA; n Rollins School of Public Health at Emory University, Atlanta, GA, USA; o Department of Infectious Diseases, Copenhagen University Hospital, Rigshospitalet, Copenhagen, Denmark; p First Department of Internal Medicine, Medical School, National and Kapodistrian University of Athens, Athens, Greece; q Unit AIDS Research Network Cohort, National Center of Epidemiology, Health Institute Carlos III, Madrid, Spain; r Stichting HIV Monitoring, Amsterdam, Netherlands; s Department of Global Health, Amsterdam University Medical Centers, University of Amsterdam, Amsterdam, Netherlands; t Amsterdam Institute for Global Health and Development, Amsterdam, Netherlands; u Yale School of Medicine, Yale University, New Haven, CT, USA; v VA Connecticut Healthcare System, West Haven, CT, USA; w Faculty of Epidemiology and Population Health, London School of Hygiene & Tropical Medicine, London, UK; x Fundación Instituto de Investigación Sanitaria Illes Balears, Infectious Diseases Unit, Hospital Son Espases, Mallorca, Spain; y Infectious Diseases Unit, Medical Center 2, Frankfurt University Hospital, Frankfurt, Germany; z Division of Research, Kaiser Permanente Northern California, Oakland, CA, USA; aa Department of Infection and Population Health, University College London, London, UK; ab Centre for Clinical Research, Epidemiology, Modelling and Evaluation, Institute for Global Health, University College London, London, UK; ac Department I of Internal Medicine, University Hospital of Cologne, Cologne, Germany; ad German Center for Infection Research, Partner Site Cologne–Bonn, Cologne, Germany; ae Division of Infectious Diseases, Department of Medicine, Vanderbilt University School of Medicine, Nashville, TN, USA

## Abstract

**Background:**

Over the past decade, antiretroviral therapy (ART) regimens that include integrase strand inhibitors (INSTIs) have become the most commonly used for people with HIV starting ART. Although trials and observational studies have compared virological failure on INSTI-based with other regimens, few data are available on mortality in people with HIV treated with INSTIs in routine care. Therefore, we compared all-cause mortality between different INSTI-based and non-INSTI-based regimens in adults with HIV starting ART from 2013 to 2018.

**Methods:**

This cohort study used data on people with HIV in Europe and North America from the Antiretroviral Therapy Cohort Collaboration (ART-CC) and UK Collaborative HIV Cohort (UK CHIC). We studied the most common third antiretroviral drugs (additional to nucleoside reverse transcriptase inhibitor) used from 2013 to 2018: rilpivirine, darunavir, raltegravir, elvitegravir, dolutegravir, efavirenz, and others. Adjusted hazard ratios (aHRs; adjusted for clinical and demographic characteristics, comorbid conditions, and other drugs in the regimen) for mortality were estimated using Cox models stratified by ART start year and cohort, with multiple imputation of missing data.

**Findings:**

62 500 ART-naive people with HIV starting ART (12 422 [19·9%] women; median age 38 [IQR 30–48]) were included in the study. 1243 (2·0%) died during 188 952 person-years of follow-up (median 3·0 years [IQR 1·6–4·4]). There was little evidence that mortality rates differed between regimens with dolutegravir, elvitegravir, rilpivirine, darunavir, or efavirenz as the third drug. However, mortality was higher for raltegravir compared with dolutegravir (aHR 1·49, 95% CI 1·15–1·94), elvitegravir (1·86, 1·43–2·42), rilpivirine (1·99, 1·49–2·66), darunavir (1·62, 1·33–1·98), and efavirenz (2·12, 1·60–2·81) regimens. Results were similar for analyses making different assumptions about missing data and consistent across the time periods 2013–15 and 2016–18. Rates of virological suppression were higher for dolutegravir than other third drugs.

**Interpretation:**

This large study of patients starting ART since the introduction of INSTIs found little evidence that mortality rates differed between most first-line ART regimens; however, raltegravir-based regimens were associated with higher mortality. Although unmeasured confounding cannot be excluded as an explanation for our findings, virological benefits of first-line INSTIs-based ART might not translate to differences in mortality.

**Funding:**

US National Institute on Alcohol Abuse and Alcoholism and UK Medical Research Council.

## Introduction

The prognosis of people with HIV treated with highly active antiretroviral therapy (ART) has improved since ART was first introduced in the mid-1990s.^1^, ^2^ Improvements have been attributed to a range of factors, including the availability of improved drug regimens that are easier to take, are less toxic, have fewer side-effects, have less potential for drug–drug interactions, and are less susceptible to resistance. These all contribute to the better adherence, potency, and durability of more modern ART regimens compared with older regimens.

The introduction of integrase strand inhibitors (INSTIs) in 2007 was an important milestone in the history of ART.^3^ Most patients now start ART with an INSTI-based regimen following positive results from randomised trials.^4^, ^5^, ^6^, ^7^, ^8^, ^9^, ^10^, ^11^, ^12^, ^13^, ^14^, ^15^, ^16^, ^17^ These trials showed superiority^7^, ^9^, ^11^, ^13^, ^14^, ^15^ or non-inferiority^6^, ^8^, ^10^, ^12^, ^16^, ^17^ of INSTI-based regimens for virological failure compared with other regimens, such as those containing efavirenz (a non-nucleoside reverse transcriptase inhibitor [NNRTI]) and atazanavir or darunavir (protease inhibitors).^6^, ^7^, ^8^, ^9^, ^10^, ^11^, ^12^, ^13^, ^14^, ^15^, ^16^, ^17^ The most commonly used INSTIs over the past decade have been raltegravir, elvitegravir, and dolutegravir, which became available in that order across North America and Europe.

Although research has been done into the incidence of virological failure on INSTI-based regimens,^18^, ^19^ there have been few reports to date on mortality in people with HIV receiving INSTIs in routine clinical care. Choice of regimen by clinicians and patients can be influenced by a number of factors, including patients’ perceived propensity to adhere, comorbidities, regimen tolerability, pill burden, and toxicity. Therefore, virological failure outcomes observed in randomised trials might not be reflected in longer term mortality patterns observed in the wider clinical population.

**Research in context**

**Evidence before this study**

We identified English language papers that studied associations between mortality in people with HIV starting different antiretroviral therapy (ART) regimens by searching PubMed with the search term “mortality HIV regimen integrase” from inception of the database to July 24, 2021. Randomised trials have found strong evidence that integrase strand inhibitor (INSTI)-based regimens (raltegravir, elvitegravir, and dolutegravir) were non-inferior or superior in terms of virological failure compared with various non-nucleotide reverse-transcriptase inhibitor (NNRTI) and protease inhibitor-based regimens. An observational study by the Centers for AIDS Research Network of Integrated Clinical Systems (CNICS) cohort found that fewer people with HIV starting ART on dolutegravir-based regimens had virological failure than those starting on other INSTI-based regimens, or non-INSTI-based regimens. The UK Collaborative HIV Cohort (UK CHIC) Study found that virological failure was more common in people with HIV starting on modern protease inhibitor-based regimens compared with modern NNRTI-based regimens. However, there has been little research on associations of modern regimens with mortality. A study of 2007–13 by the Kaiser Permanente cohort observed higher mortality for people with HIV on raltegravir than on other regimens, and a study of 2007–15 by CNICS found similar rates of AIDS-defining illness or death in people with HIV starting ART on raltegravir-based or efavirenz-based regimens. A meta-analysis of randomised trials by Kanters and colleagues, reported that low event rates restricted the quality of evidence about mortality, but found differences in rates of virological failure, with raltegravir having higher rates than most other regimens.

**Added value of this study**

Our study included 62 500 ART-naive people with HIV who started ART from 2013 to 2018 in 21 cohorts spanning 12 countries in Europe and North America. In analyses adjusted for clinical and demographic characteristics (including comorbid conditions) and other drugs in the regimen, all-cause mortality rates in people with HIV starting ART were similar for most third drugs. However, there was higher mortality in people with HIV starting ART on raltegravir-based regimens compared with dolutegravir-based (adjusted hazard ratio 1·49, 95% CI 1·15–1·94), elvitegravir-based (1·86, 1·43–2·42), rilpivirine-based (1·99, 1·49–2·66), darunavir-based (1·62, 1·33–1·98), and efavirenz-based (2·12, 1·60–2·81) regimens.

**Implications of all the available evidence**

In people with HIV starting ART between 2013 and 2018, raltegravir-based regimens were associated with higher mortality compared with other regimens, consistent with the Kaiser Permanente study, but not the CNICS study. More rapid virological suppression on dolutegravir and other integrase inhibitors might not translate into mortality benefits. However, confounding by indication cannot be excluded, although we controlled for a wide range of prognostic factors likely to influence regimen choice.

The aim of this study was to compare the prognosis of people with HIV on different INSTI-based and non-INSTI-based ART regimens, using multicountry cohort data from 2013 onwards, adjusting for potential confounding variables.

## Methods

### Study design and population

Data were combined from 21 European and North American HIV cohort studies of people with HIV from the Antiretroviral Therapy Cohort Collaboration (ART-CC)^20^ and the UK Collaborative HIV Cohort (UK CHIC; appendix p 1).^21^ Analyses were restricted to ART-naive people with HIV who started ART regimens that contained at least three drugs on or after Jan 1, 2013, when integrase inhibitor regimens became widely available, up to Dec 31, 2018 (to ensure up to 3 years potential subsequent follow-up). Eligible participants were aged 16 years or older when they started ART and had no previous exposure to ART medications. Included participants had a CD4 cell count and HIV-1 RNA viral load measurement between 1 month before and 1 week after starting ART. We excluded people with HIV who started ART with an HIV-1 RNA viral load value of 50 copies per mL or less, because they might not have been ART-naive.

Ethics committees or institutional review boards approved the individual cohorts, which used standardised data collection methods, and regularly followed up patients. Cohorts gathered information on mortality through linkage with vital statistics agencies and hospitals or physician report, and the active follow-up of participants.

### Procedures

In addition to nucleoside reverse transcriptase inhibitors (NRTIs), we studied the most frequently used third antiretroviral drugs from 2013 to 2018: rilpivirine, darunavir, raltegravir, elvitegravir, dolutegravir, efavirenz, and others. The NRTI drug pairs were stratified as: emtricitabine and tenofovir disproxil fumarate, lamivudine and abacavir, emtricitabine and tenofovir alafenamide, and others.

The demographic and biomarker variables at ART start considered in our analyses were CD4 count (cells per μL), HIV-1 RNA viral load (copies per mL), sex, age (years), HIV acquisition risk group, CD8 count (cells per μL), alanine aminotransferase (u/L), aspartate aminotransferase (u/L), haemoglobin (g/dL), creatinine (mg/dL), hepatitis C virus (HCV) RNA (positive), hepatitis B surface antigen (positive), AIDS event status (no AIDS events, had an AIDS event ever, had a tuberculosis or other mycobacterial infection in the past year, had an AIDS-defining malignancy in the past year), previous non-AIDS defining malignancy, previous cardiovascular events (acute myocardial infarctions and invasive cardiovascular procedures), and ethnicity or geographic origin amalgamated into one ethnicity variable (appendix p 1). The AIDS event status variable was created because discussions with clinicians indicated that mycobacterium, tuberculosis, or AIDS-defining malignancy might affect clinician prescribing.

HIV acquisition risk activity was categorised as men who have sex with men, injection drug use, heterosexual intercourse, and other. Ethnicity or geographic origin was categorised as White, Black, Hispanic, Asian, other, and unknown. Variables at regimen start were viral load (0–9999, 10 000–99 999, and ≥100 000 RNA copies per mL), age (16–29, 30–39, 40–49, 50–59, and ≥60 years), CD4 count (0–49, 50–99, 100–199, 200–349, 350–499, and ≥500 cells per μL), CD8 count (0–399, 400–799, 800–1199, ≥1200 cells per μL, and missing), alanine aminotransferase concentration (0–9, 10–29, 30–49, ≥50 u/L, and missing), aspartate aminotransferase (0–19, 20–39, ≥40 u/L, and missing), haemoglobin concentration (0–9, 10–14, 15–19, ≥20 g/dL, and missing), and creatinine concentration (0·0–0·49, 0·50–0·74, 0·75–0·99, ≥1·00 mg/dL, and missing). The categories for CD8 cell count, alanine aminotransferase concentration, aspartate aminotransferase concentration, haemoglobin concentration, and creatinine concentration were chosen through examination of the distribution of the data, whereas viral load and CD4 cell count were categorised as in previous ART-CC analyses. The other variables were binary variables, with an extra category for missing data when necessary.

Because the availability of demographic and biomarker variables varied across these clinical cohorts, they were grouped as either main variables that were available for all patients from all cohorts (CD4 cell count, viral load, sex, age, and AIDS event status) or additional variables (transmission group, CD8 cell count; alanine aminotransferase, aspartate aminotransferase, haemoglobin, and creatinine concentrations; HCV and hepatitis B virus positivity; previous non-AIDS-defining malignancy or cardiovascular conditions; and ethnicity or origin). Five cohorts were excluded from analyses including additional variables because their data were more than 70% unavailable for at least one additional variable.

### Statistical analysis

Hazard ratios (HRs) for all-cause mortality comparing different initial ART regimens were estimated using Cox models stratified by year of ART start and cohort using the following analyses: (1) unadjusted models; (2) adjusted for the main variables, including all cohorts; (3) adjusted for the main variables, restricted to cohorts providing additional variables; and (4) adjusted for main and additional variables, restricted to cohorts providing additional variables. Because predictors of ART regimen choice evolved rapidly between 2013 and 2018, we did additional analyses in which the association of confounders with mortality was modelled separately in two time periods: (5) as in analysis 4, separately for ART start years 2013–15 and 2016–18; and (6) inverse-variance weighted meta-analyses of HRs for 2013–15 and 2016–18 from analysis 5.

For analyses 4–6 multiple imputation was done on the variables with missing data (25 imputed datasets). The following variables required imputation: CD8 cell count; alanine aminotransferase, aspartate aminotransferase, haemoglobin, and creatinine concentrations; transmission mode; HCV positivity; HBV positivity; cardiovascular events; and ethnicity or origin. The continuous variables were log transformed before imputation, and quadratic terms of these variables were included in the imputations. Imputation was done via linear regression for numerical variables, logistic regression for binary variables, and multinomial regression for categorical variables. The variables included in each imputation regression were those included in the main and additional variable sets and ART start year (but not the regimen), death, cohort, and the Nelson–Aalen estimate of the cumulative hazard function. After imputation, the continuous variables were exponentiated and categorised as before (eg, CD8 count 0–399, 400–799, 800–1199, and ≥1200 cells per μL). Results from imputed datasets were combined using Rubin's rules.^22^ In the sensitivity analyses, we did analyses four, five, and six, but with a dummy variable for missing data instead of using multiple imputation. In a sensitivity analysis, we adjusted for potentially informative loss to follow-up by weighting the analysis by the inverse probability of loss to follow-up over time. The weights were derived by splitting the data by month of follow-up using a pooled logistic regression model with loss to follow-up as the outcome, adjusting for the main and additional variables, and cohort, year of ART start, and months after ART start (using cubic splines with 3 knots). All variables included in the logistic regression were measured at baseline.

In the analyses with mortality as the outcome, only the initial regimen was considered, and subsequent regimen switching was not accounted for in the models. We plotted Kaplan-Meier curves showing the incidence of loss to follow-up on each regimen.

In a sensitivity analysis, we investigated differences in mortality rates between starting regimens for those not presenting late for ART initiation (defined as CD4 count ≥350 cells per μL, no previous AIDS, and HIV-1 RNA viral load <100 000 copies per mL) and those presenting late for ART initiation. This analysis adjusted for the main and additional variables, restricted to cohorts providing additional variables, and used multiple imputation.

Kaplan-Meier curves were used to display the cumulative incidence of switching from the initial ART regimen up to 3 years after starting ART according to regimen. This analysis did not include UK CHIC because data on ART switches had not been requested. Data were censored after first regimen switch, loss to follow-up, administrative censoring, 3 years after ART initiation, or death, whichever occurred first. People with HIV did not have to be on a regimen for a minimum length of time to be included.

Using Fine and Gray's competing risks regression models adjusting for time to death, we investigated the time to first viral suppression after ART initiation, comparing rates between ART regimens containing different drugs. This endpoint was chosen for comparability with randomised trials comparing initial ART regimens. Viral suppression was defined as an HIV-1 RNA viral load of 50 copies per mL or less. The analysis adjusted for the main and additional variables and was restricted to cohorts providing additional variables, dummy variables were used for missingness. We also did a sensitivity analysis examining time to first virological failure, defined as the occurrence of an HIV-1 RNA viral load of 400 copies per mL or more at least 6 months after ART initiation. Analyses were done with Stata (version 16.1).

### Role of the funding source

The funders had no role in the collection, analysis or interpretation of data, report writing, or the decision to submit this study for publication.

## Results

In total, 62 500 people with HIV were included in the analyses (table 1; appendix p 2). 12 422 (19·9%) individuals were female, and the median age at the start of ART was 38 years (IQR 30–48). 162 (1·7%) people with HIV who started rilpivirine-containing regimens had a CD4 count less than 100 cells per μL, which was lower than for all other regimens. Similarly, 3072 (33·7%) people who started rilpivirine-containing regimens had viral loads less than 10 000 copies per mL, which was higher than for the other regimens. 158 (3·0%) of 5261 people who started raltegravir had previously had an non-AIDS-defining malignancy, and 243 (4·6%) had tuberculosis within a year before to starting ART, both higher than for other regimens.

**Table 1:** Patient characteristics at the time of starting antiretroviral therapy (ART), according to the third drug in the initial regimen

		**Dolutegravir (n=13 249)**	**Darunavir (n=11 322)**	**Raltegravir (n=5261)**	**Elvitegravir (n=10 673)**	**Rilpivirine (n=9120)**	**Efavirenz (n=6752)**	**Other (n=6123)**
Other drugs in antiretroviral therapy regimen								
	Emtricitabine and tenofovir disoproxil	4022 (30·4%)	9440 (83·4%)	3808 (72·4%)	6419 (60·1%)	7940 (87·1%)	6039 (89·4%)	4303 (70·3%)
	Lamivudine and abacavir	7865 (59·4%)	1358 (12·0%)	783 (14·9%)	10 (0·1%)	257 (2·8%)	486 (7·2%)	1092 (17·8%)
	Emtricitabine and tenofovir alafenamide	1112 (8·4%)	266 (2·3%)	300 (5·7%)	3722 (34·9%)	874 (9·6%)	15 (0·2%)	162 (2·6%)
	Other	250 (1·9%)	258 (2·3%)	370 (7·0%)	522 (4·9%)	49 (0·5%)	212 (3·1%)	566 (9·2%)
Sex								
	Male	11 102 (83·8%)	8347 (73·7%)	4122 (78·4%)	9163 (85·9%)	7344 (80·5%)	5908 (87·5%)	4092 (66·8%)
	Female	2147 (16·2%)	2975 (26·3%)	1139 (21·6%)	1510 (14·1%)	1776 (19·5%)	844 (12·5%)	2031 (33·2%)
Age (years)								
	16–29	3143 (23·7%)	2583 (22·8%)	1085 (20·6%)	2769 (25·9%)	2272 (24·9%)	1559 (23·0%)	1600 (26·1%)
	30–39	3714 (28·0%)	3504 (30·9%)	1511 (28·7%)	3199 (30·0%)	2997 (32·9%)	2112 (31·3%)	2108 (34·4%)
	40–49	3226 (24·3%)	2894 (25·6%)	1369 (26·0%)	2550 (23·9%)	2292 (25·1%)	1681 (24·9%)	1442 (23·6%)
	50–59	2197 (16·6%)	1620 (14·3%)	839 (15·9%)	1559 (14·6%)	1163 (12·8%)	974 (14·4%)	720 (11·8%)
	≥60	969 (7·3%)	721 (6·4%)	457 (8·7%)	596 (5·9%)	396 (4·3%)	426 (6·3%)	253 (4·1%)
HIV risk activity								
	Sex between men	7486 (56·5%)	4819 (42·6%)	2607 (49·6%)	6185 (57·9%)	5208 (57·1%)	3702 (54·8%)	2657 (43·4%)
	Injecting drug use	411 (3·1%)	411 (3·6%)	196 (3·7%)	268 (2·5%)	343 (3·8%)	166 (2·5%)	266 (4·3%)
	Heterosexual sex	3758 (28·4%)	4994 (44·1%)	1785 (33·9%)	2803 (26·3%)	2789 (30·6%)	1697 (25·1%)	2534 (41·4%)
	Other	1594 (12·0%)	1098 (9·7%)	673 (12·8%)	1417 (13·3%)	780 (8·6%)	1187 (17·7%)	666 (10·9%)
CD4 count (cells per μL)								
	0–49	1231 (9·3%)	1654 (14·6%)	641 (12·2%)	616 (5·8%)	70 (0·8%)	483 (7·2%)	641 (10·7%)
	50–99	764 (5·8%)	980 (8·7%)	436 (8·3%)	435 (4·1%)	92 (1·0%)	314 (4·7%)	377 (6·2%)
	100–199	1345 (10·2%)	1681 (14·8%)	683 (13·0%)	1019 (9·5%)	434 (4·8%)	730 (10·8%)	815 (13·3%)
	200–349	2741 (20·7%)	2593 (22·9%)	1071 (20·4%)	2266 (21·2%)	1936 (21·2%)	1658 (24·6%)	1473 (24·1%)
	350–499	2964 (22·4%)	2171 (19·2%)	1063 (20·2%)	2724 (25·5%)	2864 (31·4%)	1773 (26·3%)	1407 (23·0%)
	≥500	4204 (31·7%)	2243 (19·8%)	1367 (26·0%)	3613 (33·9%)	3724 (40·8%)	1794 (26·7%)	1410 (23·0%)
HIV-1 RNA viral load (copies per mL)								
	0–9999	2317 (17·5%)	1516 (13·4%)	853 (16·2%)	2081 (19·5%)	3072 (33·7%)	1158 (17·2%)	1365 (22·3%)
	10 000–99 999	5263 (39·7%)	3891 (34·4%)	1860 (35·4%)	4654 (43·6%)	5559 (61·0%)	2784 (41·2%)	2450 (40·0%)
	≥100 000	5669 (42·8%)	5915 (52·2%)	2548 (48·4%)	3938 (36·9%)	489 (5·4%)	2810 (41·6%)	2308 (37·7%)
Presenting late*		8288 (62·6%)	8618 (76·1%)	3771 (71·7%)	6109 (57·2%)	2926 (32·1%)	4477 (66·3%)	4104 (67·0%)

* Not presenting late was defined as starting ART with a CD4 count of 350 cells per μL, no previous AIDS, and a viral load less than 100 000 copies per mL.

The most common ART drug was dolutegravir, which was used by 13 349 (21·2%) people (table 2; appendix p 4). The proportion of people with HIV who started ART with elvitegravir and dolutegravir as the third drugs in their ART regimen increased between 2013 and 2018, whereas the proportion of people with rilpivirine, efavirenz, and other regimens decreased in the same period (table 2). 29 925 (78·2%) of 38 285 regimens started in 2013–15 had emtricitabine and tenofovir disproxil fumarate as the other components, but this dropped to 12 046 (49·7%) of 24215 from 2016 to 2018 (appendix p 4). Emtricitabine and tenofovir alafenamide were the additional regimen components for 1200 (3·1%) people in 2013–15, increasing to 5251 (21·7%) in 2016–18. 5567 (14·5%) of regimens started in 2013–15 included lamivudine and abacavir, increasing to 6284 (26·0%) of regimens in 2016–18.

**Table 2:** Number of people starting on each regimen, rates of deaths, and numbers of deaths by ART start regimen type for all cohorts

	**ART start 2013–18**	**ART start 2013–15**	**ART start 2016–18**	**Median (IQR) follow-up (years)**
**Overall**				
Started regimen	62 500 (100%)	38 285 (100%)	24 215 (100%)	3·0 (1·6–4·4)
Deaths	1243 (2·0%)	961 (2·5%)	282 (1·2%)	..
Mortality rate per 1000 years (95% CI)	6·6 (6·2–7·0)	6·4 (6·0–6·8)	7·2 (6·4–8·1)	..
**Dolutegravir**				
Started regimen	13 249 (21·2%)	3876 (10·1%)	9373 (38·7%)	1·9 (1·1–2·9)
Deaths	208 (1·6%)	88 (2·3%)	120 (1·3%)	..
Mortality rate per 1000 years (95% CI)	7·7 (6·7–8·8)	7·2 (5·9–8·9)	8·0 (6·7–9·6)	..
**Darunavir**				
Started regimen	11 322 (18·1%)	7840 (20·5%)	3482 (14·4%)	3·6 (1·9–4·9)
Deaths	294 (2·6%)	237 (3·0%)	57 (1·6%)	..
Mortality rate per 1000 years (95% CI)	7·7 (6·9–8·6)	7·3 (6·4–8·3)	10·0 (7·7–13·0)	..
**Raltegravir**				
Started regimen	5261 (8·4%)	3355 (8·8%)	1906 (7·9%)	2·8 (1·5–4·4)
Deaths	232 (4·4%)	189 (5·6%)	43 (2·3%)	..
Mortality rate per 1000 years (95% CI)	15·0 (13·2–17·1)	15·0 (13·0–17·3)	15·0 (11·1–20·2)	..
**Elvitegravir**				
Started regimen	10 673 (17·1%)	5038 (13·2%)	5635 (23·3%)	2·4 (1·4–3·7)
Deaths	129 (1·2%)	91 (1·8%)	38 (0·7%)	..
Mortality rate per 1000 years (95% CI)	4·8 (4·0–5·6)	5·1 (4·2–6·3)	4·1 (3·0–5·6)	..
**Rilpivirine**				
Started regimen	9120 (14·6%)	6988 (18·3%)	2132 (8·8%)	3·9 (2·2–5·0)
Deaths	95 (1·0%)	89 (1·3%)	6 (0·3%)	..
Mortality rate per 1000 years (95% CI)	2·9 (2·4–3·5)	3·1 (2·5–3·8)	1·7 (0·7–3·7)	..
**Efavirenz**				
Started regimen	6752 (10·8%)	6081 (15·9%)	671 (2·8%)	4·2 (2·8–5·1)
Deaths	131 (1·9%)	128 (2·1%)	3 (0·4%)	..
Mortality rate per 1000 years (95% CI)	5·0 (4·2–6·0)	5·1 (4·3–6·1)	2·8 (0·9–8·7)	..
**Others**				
Started regimen	6123 (9·8%)	5107 (13·3%)	1016 (4·2%)	3·9 (2·2–5·0)
Deaths	154 (2·5%)	139 (2·7%)	15 (1·5%)	..
Mortality rate per 1000 years	6·9 (5·9–8·1)	6·7 (5·7–8·0)	9·7 (5·8–16·1)	..

Data are n (%), unless otherwise stated. ART=antiretroviral therapy.

The cumulative proportions of switching by 3 years after starting ART were highest for people who started on efavirenz, raltegravir, and darunavir regimens (all with <50% remaining on the regimens), and were lowest for people who started dolutegravir, elvitegravir, and rilpivirine regimens (all with approximately 75% remaining on the regimens; figure).

**Figure:**
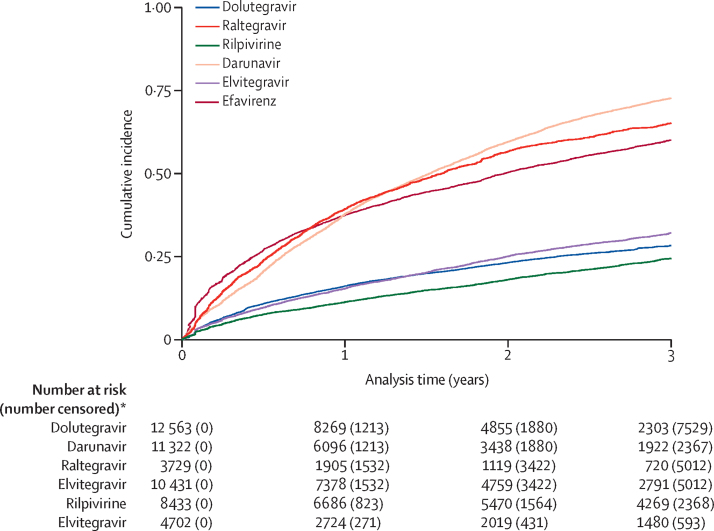
Kaplan-Meier estimates of the cumulative incidence of regimen switching *People were censored at death, loss to follow-up, or administrative censoring, whichever occurred first.

Overall, 1243 (2·0%) of the 62 500 participants died after 188 952 person-years follow-up (median follow-up 3·0 years [IQR 1·6–4·4]). The mortality rate per 1000 person-years was 6·6 (95% CI 6·2–7·0). The median follow-up time varied from 1·9 years (IQR 1·1–2·9) for dolutegravir to 4·2 years (2·8–5·1) for elvitegravir. The mortality rates per 1000 person-years were 15·0 (95% CI 13·2–17·1) for raltegravir, 7·7 (6·9–8·6) for darunavir, 7·7 (6·7–8·8) for dolutegravir, 6·9 (5·9–8·1) for other regimens, 5·0 (4·2–6·0) for efavirenz, 4·8 (4·0–5·6) for elvitegravir, and 2·9 (2·4–3·5) for rilpivirine (table 2). Loss to follow-up was lowest in those on efavirenz-based regimens, but otherwise broadly similar across the regimens (appendix p 5).

Associations between ART regimen and all-cause mortality showed that adjusted hazard ratios (aHRs) were generally substantially attenuated towards the null compared with crude HRs (table 3). Estimated HRs did not change substantially after additional restriction to the cohorts providing additional variables, when additionally adjusting for the additional variables, or when meta-analysing the analyses across time periods. Results were also similar in sensitivity analyses using dummy variables when data were missing, instead of using multiple imputation (appendix p 6). In analyses weighted by the inverse probability of loss to follow-up, the association of raltegravir-based regimens with higher mortality compared with other regimens remained, although most odds ratios comparing other pairs of regimens were attenuated towards 1 (appendix p 7).

**Table 3:** Hazard ratios for mortality for each third drug comparison

	**All (n=62 500)**		**Those providing additional variables (n=50 722)**			**Those providing additional variables**		
	Unadjusted analysis	Adjusted for main variables*	Adjusted for main variables†	Adjusted for additional variables‡	Meta-analysed across time periods for additional variables§	Adjusted for additional variables, 2013–15 (n=29 989)¶	Adjusted for additional variables, 2016–18 (n=20 733)‖	p value 2013–15 *vs* 2016–18
Rilpivirine *vs* dolutegravir	0·45 (0·34–0·58)	0·77 (0·57–1·02)	0·74 (0·54–1·01)	0·83 (0·61–1·13)	0·78 (0·55–1·10)	0·82 (0·57–1·20)	0·58 (0·24–1·37)	0·47
Darunavir *vs* dolutegravir	1·19 (0·97–1·47)	0·96 (0·77–1·20)	0·92 (0·73–1·15)	0·96 (0·76–1·21)	0·98 (0·77–1·25)	0·94 (0·69–1·27)	1·05 (0·71–1·55)	0·67
Raltegravir *vs* dolutegravir	2·32 (1·87–2·88)	1·79 (1·42–2·26)	1·73 (1·35–2·21)	1·60 (1·26–2·05)	1·49 (1·15–1·94)	1·62 (1·18–2·23)	1·27 (0·81–1·99)	0·39
Elvitegravir *vs* dolutegravir	0·57 (0·45–0·72)	0·80 (0·62–1·03)	0·74 (0·57–0·96)	0·84 (0·65–1·10)	0·79 (0·60–1·05)	0·88 (0·62–1·25)	0·66 (0·41–1·05)	0·34
Efavirenz *vs* dolutegravir	0·62 (0·48–0·81)	0·68 (0·52–0·90)	0·70 (0·52–0·94)	0·78 (0·58–1·04)	0·75 (0·53–1·07)	0·78 (0·55–1·12)	0·19 (0·02–1·46)	0·20
Rilpivirine *vs* elvitegravir	0·78 (0·60–1·03)	0·96 (0·72–1·27)	1·00 (0·74–1·35)	0·99 (0·73–1·33)	0·93 (0·68–1·28)	0·94 (0·67–1·31)	0·88 (0·36–2·17)	0·89
Darunavir *vs* elvitegravir	2·09 (1·67–2·60)	1·20 (0·96–1·51)	1·24 (0·98–1·58)	1·14 (0·90–1·45)	1·17 (0·92–1·50)	1·06 (0·80–1·41)	1·60 (0·97–2·64)	0·16
Raltegravir *vs* elvitegravir	4·06 (3·23–5·09)	2·24 (1·77–2·83)	2·35 (1·83–3·01)	1·91 (1·48–2·46)	1·86 (1·43–2·42)	1·84 (1·37–2·49)	1·94 (1·12–3·37)	0·87
Efavirenz *vs* elvitegravir	1·09 (0·84–1·42)	0·86 (0·65–1·12)	0·95 (0·71–1·27)	0·92 (0·69–1·23)	0·87 (0·64–1·18)	0·89 (0·65–1·21)	0·29 (0·04–2·29)	0·28
Darunavir *vs* rilpivirine	1·91 (1·53–2·39)	1·26 (0·98–1·62)	1·24 (0·95–1·62)	1·16 (0·89–1·52)	1·19 (0·91–1·57)	1·14 (0·85–1·51)	1·83 (0·76–4·40)	0·32
Raltegravir *vs* rilpivirine	5·17 (4·06–6·58)	2·34 (1·81–3·04)	2·34 (1·77–3·10)	1·93 (1·46–2·57)	1·99 (1·49–2·66)	1·97 (1·45–2·67)	2·21 (0·89–5·25)	0·81
Efavirenz *vs* rilpivirine	1·39 (1·06–1·83)	0·89 (0·67–1·19)	0·94 (0·69–1·28)	0·94 (0·69–1·27)	0·93 (0·68–1·27)	0·95 (0·69–1·30)	0·33 (0·04–2·93)	0·34
Raltegravir *vs* darunavir	1·95 (1·62–2·33)	1·86 (1·55–2·24)	1·89 (1·55–2·30)	1·67 (1·37–2·04)	1·62 (1·33–1·98)	1·73 (1·39–2·16)	1·21 (0·76–1·94)	0·18
Efavirenz *vs* darunavir	0·52 (0·42–0·65)	0·71 (0·56–0·89)	0·76 (0·59–0·98)	0·81 (0·63–1·04)	0·82 (0·63–1·07)	0·84 (0·64–1·09)	0·18 (0·02–1·39)	0·16
Raltegravir *vs* efavirenz	3·72 (2·98–4·65)	2·62 (2·07–3·32)	2·48 (1·91–3·23)	2·09 (1·60–2·73)	2·12 (1·60–2·81)	2·07 (1·56–2·76)	6·67 (0·86–42·2)	0·24

Data are hazard ratio (95% CI). Multiple imputation to account for missing data among the additional variables.

* Adjusted for the main variables, including all cohorts.

† Adjusted for the main variables, restricted to cohorts providing additional variables.

‡ Adjusted for main and additional variables, restricted to cohorts providing additional variables.

§ Overall inverse-variance weighted meta-analyses of adjusted hazard ratios.

¶ Adjusted hazard ratios for those starting ART in 2013–15.

‖ Adjusted hazard ratios for those starting ART in 2016–18.

In the meta-analyses across time periods (the most completely adjusted analyses), there was little evidence of lower mortality between rilpivirine-containing regimens and dolutegravir-containing ART (aHR 0·78, 95% CI 0·55–1·10; table 3). Mortality was similar between darunavir and dolutegravir (0·98, 0·77–1·25). There was little evidence of differences in mortality between rilpivirine-containing and elvitegravir-containing ART (0·93, 0·68–1·20), or between efavirenz-based and elvitegravir-based regimens (0·87, 0·64–1·18). aHRs comparing elvitegravir with dolutegravir, efavirenz with dolutegravir, darunavir with elvitegravir, and darunavir with rilpivirine, efavirenz with rilpivirine, and efavirenz with darunavir were all in the range 0·75–1·19 (table 3).

Mortality was higher when starting raltegravir-containing ART compared with dolutegravir-containing ART (aHR 1·49, 95% CI 1·15–1·94) and elvitegravir-containing ART (1·86, 1·43–2·42). Mortality was also higher when starting raltegravir-containing ART compared with rilpivirine-containing (2·00, 1·50–2·67), efavirenz-containing (2·12, 1·60–2·81), and darunavir-containing (1·62, 1·33–1·98) ART.

In people with HIV in cohorts that provided both main and additional variables, 24 690 (48·7%) of 50 722 people presented late for treatment. For patients who did and did not present late, HRs were generally attenuated after adjusting for patient characteristics (main and additional variables) at the time of starting ART (appendix p 8). Adjusted mortality HRs comparing raltegravir-containing ART with other regimens were consistently higher in patients who did not present late (ranging from 2·74 [95% CI 1·62–4·64] compared with darunavir-containing regimens to 3·24 [1·91–5·48] compared with rilpivirine-containing regimens; appendix p 8) than those in patients who presented late (ranging from 1·49 [1·14–1·94] for dolutegravir-containing regimens to 1·97 [1·46–2·67] for efavirenz-containing regimens; appendix p 8). aHRs for comparisons between other third drugs were similar in patients who did and did not present late.

Of the 50 722 people with HIV included in the cohorts providing both main and additional variables, 45 037 (88·8%) had viral suppression, and 1081 (2·1%) died before viral suppression. Rates of viral suppression were lower (longer time to viral suppression) for all third drugs compared with dolutegravir, and rates of viral suppression were lower (longer time to suppression) for all regimens except dolutegravir compared with elvitegravir (appendix p 9). People with HIV on raltegravir-containing regimens and efavirenz-containing regimens had faster time to viral suppression than those on rilpivirine-containing regimens and darunavir-containing regimens, and people with HIV on raltegravir-containing regimens had faster time to suppression than those on efavirenz-containing regimens. Rates of suppression were similar for people with HIV on rilpivirine and darunavir. Of the 51 837 people with HIV who survived to 6 months after starting ART, virological failure was recorded in 6106 (12·0%) people. 383 (0·8%) people died before 6 months. People with HIV who started ART on dolutegravir-containing regimens had lower rates of virological failure compared with all other regimens, and people on raltegravir-containing and efavirenz-containing regimens had higher rates compared with people with HIV on elvitegravir-containing and rilpivirine-containing regimens (appendix p 10). People with HIV on darunavir-containing regimens had higher rates of virological failure compared with people with HIV on rilpivirine-containing regimens.

## Discussion

In analyses adjusting for a wide-range of variables at baseline, there was no strong evidence of differences in rates of all-cause mortality between people with HIV starting ART, since the introduction of INSTIs. However, starting ART on raltegravir-based regimens was associated with higher mortality than starting on dolutegravir-based, elvitegravir-based, rilpivirine-based, darunavir-based, and efavirenz-based regimens. The proportion of people switching from their initial ART regimen within 3 years were highest for those who started ART on efavirenz, raltegravir, and darunavir, and were lowest for dolutegravir, elvitegravir, and rilpivirine. Rates of viral suppression were highest for dolutegravir-based and elvitegravir-based regimens, and higher for raltegravir-based regimens than for rilpivirine-based, darunavir-based, and efavirenz-based regimens.

To our knowledge, this is the first multicountry study in Europe or North America to examine associations between starting ART regimen type and mortality in people with HIV in the era of INSTI regimens. A study of the Kaiser Permanente cohort for the 2007–13 time period from Horberg and colleagues^23^ also found that raltegravir-based regimens were associated with higher mortality (aHR 1·53, 95% CI 1·02–2·31) than other regimens, although this analysis included people with HIV receiving raltegravir as a second-line or third-line regimen. When restricting the analysis to people only receiving first-line ART, the aHR was 1·63 (0·82–3·24).^23^ Horberg and colleagues^23^ also found higher incidence of AIDS-defining malignancies, non-AIDS defining malignancies, and lipodystrophy in people with HIV receiving raltegravir. A study by the Centers for AIDS Research Network of Integrated Clinical Systems (CNICS) cohort found similar rates of AIDS-defining illness or death comparing raltegravir-based regimens with efavirenz-based regimens.^24^ A meta-analysis of randomised trials comparing first-line ART regimens found differing rates of viral suppression, including lower rates for raltegravir-based regimens than others, but low event rates restricted the quality of evidence for between-regimen differences in mortality.^25^ A study of data from North American cohorts by Lu and colleagues^26^ found that the risk of a composite endpoint of AIDS, acute myocardial infarction, stroke, end-stage renal disease, end-stage liver disease, or death was similar for participants whose first ART regimen was INSTI-based and efavirenz-based.^26^ That study included raltegravir, but did not compare raltegravir-based regimens separately with efavirenz-based regimens.

There is substantial evidence on associations between initial ART regimen and virological outcomes. An intention-to-treat analysis from the UK CHIC study found adjusted risk ratios for virological failure of 1·18 (95% CI 0·98–1·42) for people with HIV starting ART on INSTI-based regimens compared with NNRTI-based regimens, and 1·83 (1·61–2·08) for protease inhibitor-based regimens compared with NNRTI-based regimens.^18^ A 2013–17 study by the CNICS cohort also found that rates of virological failure were lower in people with HIV starting ART on dolutegravir-based regimens (7%) compared with other INSTI-based regimens (12%) and darunavir-based regimens (28%).^19^ Our study also showed that people with HIV on dolutegravir-containing regimens had lower rates of virological failure compared with other regimens, whereas people with HIV on raltegravir-containing and efavirenz-containing regimens had higher rates of virological failure compared with elvitegravir-containing and rilpivirine-containing regimens.

Our study uses data on 62 500 people with HIV who started ART between 2013 and 2018 in 12 countries in Europe and North America, so should be generalisable to outcomes in adults with HIV starting ART in high-income countries. 80% of our study population were men, and data on pregnancy in women were not available. We adjusted for 19 potentially confounding variables that could have influenced clinician decision making, and we dealt with missing data in these variables by restricting analyses and the use of multiple imputation. However, our results might have been affected by residual or unmeasured confounding. Decisions about the use of specific ART regimens were made by clinicians and patients, and could have been based on factors beyond those adjusted for in these analyses, such as perceived propensity to adhere to the prescribed regimen. Differing drug half-lives might influence regimens prescribing to patients for whom clinicians doubt their ability to adhere to ART, such as those with a history of substance use. Furthermore, several potentially confounding variables were not routinely collected in many of the included cohorts, including cholesterol, thrombocytes and platelets, previous end-stage renal disease, chronic obstructive pulmonary disease, recent hospitalisations, recent smoking, alcohol consumption, recent injection drug use, and recent non-injecting drug use. It is possible that people with HIV with worse prognosis, beyond the factors adjusted for in our study, were more likely to start ART on raltegravir. For example, a higher percentage of those who started on raltegravir previously had non-AIDS-defining malignancy, HCV, and tuberculosis, possibly due to worries about drug–drug interactions.^27^ Because raltegravir was the first drug in its class, there was substantial research into its interactions with other drugs, and clinicians might have been more likely to choose it for people with HIV who have comorbidities, compared with other regimens. Conversely, elvitegravir combined with cobicistat is contraindicated for people with HIV taking many medications, so it might have been prescribed less in those with comorbid conditions.^28^

Because there might have been differences in prescribing, reporting of outcomes, and health-care practices across cohorts, countries, and regions,^29^ we stratified analyses by both cohort and ART start year. More than half of the ART-CC cohorts have linkage with national death registries and several other cohorts link to local death registries^29^ to ascertain deaths in patients otherwise lost to follow-up. Several cohorts have procedures in place to contact and track patients that have been lost to follow-up. We did not have enough information available to include bictegravir in these analyses.^30^ We chose to not censor at regimen change, so that estimated associations correspond to intention-to-treat estimates from clinical trials. Rates of regimen switching within 3 years of ART start varied between regimens, with switching being more common for older ART regimens, such as raltegravir, darunavir, and efavirenz. We do not have information on the reasons for these switches, so are unable to comment on whether patients had to change regimen due to adverse events, such as immune reconstitution inflammatory syndrome (IRIS) a phenomenon related to morbidity and mortality,^31^ or whether the reasons for these switches differed between regimens. Finally, this study uses data from adults with HIV, so its findings might not be generalisable to children.

We found little evidence for differences in rates of mortality between most first-line ART regimens in the era of INSTIs. However, starting ART on raltegravir-based regimens was associated with higher mortality than on other regimens, although this could be due to unmeasured confounding. The percentage of people with HIV starting ART on raltegravir remained low between the two ART start periods studied (9% during 2013–15 to 8% during 2016–18), but these results might imply that other regimens are preferred unless there are clear reasons to choose raltegravir. The overall trend was of an increased use of INSTI-based regimens, particularly dolutegravir and elvitegravir, with another integrase inhibitor, bictegravir, coming into use at the end of this period.^30^ Our study suggests that virological advantages of these regimens do not necessarily translate to lower mortality.

## Data sharing

Due to the data sharing agreements between individual cohorts and Antiretroviral Therapy Cohort Collaboration, the data collected for this study cannot be shared. Data are owned by the individual cohorts, and those wishing to access these data should contact the individual cohorts, details of which are given in the appendix (p 1).

## Declaration of interests

MC reports grants and payments for expert testimony from Gilead, Merck, and ViiV Healthcare, paid to their institution; and payment support for attending meetings from Gilead. PC reports advisory board and expert fees from ViiV Healthcare, Gilead, and Merck; lecture fees from ViiV Healthcare and Gilead; and travel grants paid to their institution from ViiV Healthcare and Gilead. CS reports fees from Gilead for an educational presentation. MJG reports honoraria in the last 3 years from ad hoc membership of national HIV advisory boards, and from Merck, Gilead, and ViiV Healthcare. MP reports honoraria for presentations from Gilead and Merck; consulting fees from Gilead, Merck, and AbbVie; and a research grant from Gilead. FB reports travel grants and honoraria from ViiV Healthcare, Gilead, Janssen, and Merck; consulting fees and payment for expert testimony from Gilead; and support for attending meetings from Gilead, Janssen, Merck, and ViiV Healthcare. PR reports independent scientific grant support from Gilead Sciences, Merck, and ViiV Healthcare, paid to their institution; and has served on scientific advisory boards for Gilead Sciences, ViiV Healthcare, and Merck, for which honoraria were all paid to their institution, none related to the content of this Article. MRi reports funds from Gilead for attending meetings; and payment for lectures from ViiV Healthcare. MRa reports grants from Gilead. GB reports consulting fee from MedIQ; payments and honoraria from the University of Kentucky, Lexington, Kentucky, and StateServ; and funding from Merck, Eli Lily, Kaiser Permanente, and Amgen paid to their institution. HC reports research grant funding from ViiV Healthcare, National Institute of Health, and Agency for Healthcare Research and Quality, paid to their institution; and sits on the National Institute of Health Office of AIDS Research Advisory Council. NO reports funding from the Preben og Anne Simonsens Fond. CAS reports honoraria from Gilead Sciences, ViiV Healthcare, and Janssen–Cilag for membership of Data Safety and Monitoring Boards, Advisory Boards, and for preparation of educational materials; and is the vice-chair of the British HIV Association. PD reports consulting fees from Gilead, Merck, Janssen and Cilag, ViiV Healthcare, Roche, and Theratechnologies; and payment or honoraria for lectures from Gilead, Merck, Janssen, ViiV Healthcare, and Roche. All other authors declare no competing interests.
