# Supplementary material for: Associations of modern initial antiretroviral drug regimens with all-cause mortality in adults with HIV in Europe and North America: a cohort study
Source: Lancet HIV. 2022 May 31;9(6):e404–13. doi: 10.1016/S2352-3018(22)00046-7 (PMC9647005; doi:10.1016/S2352-3018(22)00046-7)
Supplement: Supplementary appendix [file mmc1.pdf]

THE LANCET
HIV

Supplementary appendix

This appendix formed part of the original submission and has been peer reviewed. We post it as supplied by the authors.

Supplement to: Trickey A, Zhang L, Gill MJ, et al. Associations of modern initial antiretroviral drug regimens with all-cause mortality in adults with HIV in Europe and North America: a cohort study. *Lancet HIV* 2022; **6:** e404–13.

Associations of modern initial antiretroviral drug regimens with all-cause mortality in adults with HIV in Europe and North America: a cohort study

Supplementary information

2022/04/27

Adam TRICKEY, Lei ZHANG, M. John GILL, Fabrice BONNET, Greer BURKHOLDER, Antonella CASTAGNA, Matthias CAVASSINI, Piotr CICHON, Heidi CRANE, Pere DOMINGO, Sophie GRABAR, Jodie GUEST, Niels OBEL, Mina PSICHOGIOU, Marta RAVA, Peter REISS, Christopher T. RENTSCH, Melchor RIERA, Gundolf SCHUETTFORT, Michael J. SILVERBERG, Colette SMITH, Melanie STECHER, Timothy R. STERLING, Suzanne M. INGLE, Caroline A. SABIN, Jonathan A.C. STERNE

Cohorts included:

AHIVCOS, AMACS, ATHENA, Alberta, Aquitaine, CBC, CoRIS, DHK, FHDH, Frankfurt, ICONA, KP, PISCIS, SHCS, UAB, UK CHIC Study (including Royal Free), UW, VACH, VACS, Vanderbilt.

See here for further information:

ART-CC: http://www.bristol.ac.uk/art-cc/whoswho/
UK CHIC: https://www.ucl.ac.uk/global-health/research/z-research/uk-collaborative-hiv-cohort-uk-chic-study

Combining ethnicity and origin variables to create a single ethnicity variable:

As cohorts had differing data availability relating to ethnicity, to reduce missingness, a new variable was created that also contained information on country of origin as a proxy marker for ethnicity. Where a cohort had data on ethnicity available then this information was used and categorised into groups (White, Black, Asian, Hispanic, Other, Unknown). Where a cohort had no data available on ethnicity, but had data available on origin, then data on origin would be used for this new variable. The ethnicity would be derived as the majority ethnicity of the country of origin (referring to Wikipedia). For countries where there was no majority ethnicity (e.g., Suriname where the two largest ethnic groups are 27% Indian, 22% Maroon-Bushinengue) then the ethnicity would be coded as unknown. Where data on both origin and ethnicity were missing then the ethnicity would be coded as unknown.

Supplementary table 1: Patient characteristics at the time of starting antiretroviral therapy (ART), according to the third drug in the initial regimen. Percentages are column percentages within variable categories, except for row percentages corresponding to the totals on each third drug.

Variable		DTG	DRV	RAL	EVG	RPV	EFV	OTH	Total
**CD8 count (cells/µL)**	0-399	1228 (9%)	1391 (12%)	590 (11%)	671 (6%)	329 (4%)	401 (6%)	517 (8%)	5127 (8%)
	400-799	3702 (28%)	3417 (30%)	1398 (27%)	2953 (28%)	2638 (29%)	1571 (23%)	1722 (28%)	17401 (28%)
	800-1199	3214 (24%)	2680 (24%)	1227 (23%)	2628 (25%)	2599 (28%)	1555 (23%)	1462 (24%)	15365 (25%)
	≥1200	3070 (23%)	2422 (21%)	1244 (24%)	2669 (25%)	2248 (25%)	1688 (25%)	1321 (22%)	14662 (23%)
	Missing	2035 (15%)	1412 (12%)	802 (15%)	1752 (16%)	1306 (14%)	1537 (23%)	1101 (18%)	9945 (16%)
**Alanine aminotransferase (u/L)**	0-9	160 (1%)	118 (1%)	76 (1%)	116 (1%)	93 (1%)	61 (1%)	118 (2%)	742 (1%)
	10-29	6011 (45%)	4717 (42%)	1803 (34%)	4793 (45%)	4312 (47%)	2501 (37%)	2450 (40%)	26587 (43%)
	30-49	2644 (20%)	2055 (18%)	973 (18%)	2042 (19%)	1582 (17%)	1248 (18%)	923 (15%)	11467 (18%)
	≥50	1927 (15%)	1594 (14%)	838 (16%)	1285 (12%)	732 (8%)	738 (11%)	651 (11%)	7765 (12%)
	Missing	2507 (19%)	2838 (25%)	1571 (30%)	2437 (23%)	2401 (26%)	2204 (33%)	1981 (32%)	15939 (26%)
**Aspartate aminotransferase (u/L)**	0-19	1638 (12%)	1333 (12%)	425 (8%)	1310 (12%)	1315 (14%)	485 (7%)	608 (10%)	7114 (11%)
	20-39	5929 (45%)	5099 (45%)	1416 (27%)	4907 (46%)	3926 (43%)	1844 (27%)	1593 (26%)	24714 (40%)
	≥40	1972 (15%)	1875 (17%)	776 (15%)	1342 (13%)	717 (8%)	586 (9%)	472 (8%)	7740 (12%)
	Missing	3710 (28%)	3015 (27%)	2644 (50%)	3114 (29%)	3162 (35%)	3837 (57%)	3450 (56%)	22932 (37%)
**Haemoglobin (g/dL)**	0-9	763 (6%)	856 (8%)	380 (7%)	315 (3%)	255 (3%)	258 (4%)	310 (5%)	3137 (5%)
	10-14	5716 (43%)	4532 (40%)	2313 (44%)	4329 (41%)	3401 (37%)	2626 (39%)	2491 (41%)	25408 (41%)
	15-19	2671 (20%)	1290 (11%)	728 (14%)	2159 (20%)	1923 (21%)	1058 (16%)	698 (11%)	10527 (17%)
	≥20	17 (0%)	2 (0%)	12 (0%)	12 (0%)	11 (0%)	75 (1%)	106 (2%)	235 (0%)
	Missing	4082 (31%)	4642 (41%)	1828 (35%)	3858 (36%)	3530 (39%)	2735 (41%)	2518 (41%)	23193 (37%)
**Creatinine (mg/dL)**	0-0.4	84 (1%)	69 (1%)	77 (1%)	31 (0%)	18 (0%)	41 (1%)	158 (3%)	478 (1%)
	0.5-0.74	1723 (13%)	806 (7%)	942 (18%)	1082 (10%)	916 (10%)	1069 (16%)	1151 (19%)	7689 (12%)
	0.75-0.99	4177 (32%)	1467 (13%)	1228 (23%)	3070 (29%)	2194 (24%)	1939 (29%)	1398 (23%)	15473 (25%)
	≥1	2088 (16%)	738 (7%)	561 (11%)	1509 (14%)	1002 (11%)	1016 (15%)	476 (8%)	7390 (12%)
	Missing	5177 (39%)	8242 (73%)	2453 (47%)	4981 (47%)	4990 (55%)	2687 (40%)	2940 (48%)	31470 (50%)
**AIDS**	None	11732 (89%)	9699 (86%)	4334 (82%)	9857 (92%)	8856 (97%)	5933 (88%)	5488 (90%)	55899 (89%)
	Prior AIDS	1220 (9%)	1334 (12%)	597 (11%)	703 (7%)	209 (2%)	541 (8%)	521 (9%)	5125 (8%)
	Recent mycobacterium	25 (0%)	24 (0%)	20 (0%)	7 (0%)	1 (0%)	17 (0%)	12 (0%)	106 (0%)
	Recent TB	152 (1%)	82 (1%)	243 (5%)	25 (0%)	14 (0%)	188 (3%)	32 (1%)	736 (1%)
	Recent ADM	120 (1%)	183 (2%)	67 (1%)	81 (1%)	40 (0%)	73 (1%)	70 (1%)	634 (1%)
**HBsAg status**	Negative	10783 (81%)	8479 (75%)	4253 (81%)	8381 (79%)	7303 (80%)	5475 (81%)	5119 (84%)	49793 (80%)
	Positive	215 (2%)	371 (3%)	150 (3%)	351 (3%)	261 (3%)	181 (3%)	160 (3%)	1689 (3%)
	Missing	2251 (17%)	2472 (22%)	858 (16%)	1941 (18%)	1556 (17%)	1096 (16%)	844 (14%)	11018 (18%)

Variable		DTG	DRV	RAL	EVG	RPV	EFV	OTH	Total
**HCV RNA status**	Negative	10609 (80%)	8956 (79%)	4191 (80%)	8742 (82%)	7264 (80%)	5907 (87%)	5127 (84%)	50796 (81%)
	Positive	571 (4%)	451 (4%)	345 (7%)	342 (3%)	413 (5%)	279 (4%)	303 (5%)	2704 (4%)
	Missing	2069 (16%)	1915 (17%)	725 (14%)	1589 (15%)	1443 (16%)	566 (8%)	693 (11%)	9000 (14%)
**NADM**	No	12263 (93%)	11101 (98%)	3563 (68%)	10226 (96%)	8353 (92%)	4277 (63%)	3849 (63%)	53632 (86%)
	Yes	221 (2%)	126 (1%)	158 (3%)	103 (1%)	71 (1%)	74 (1%)	38 (1%)	791 (1%)
	Missing	765 (6%)	95 (1%)	1540 (29%)	344 (3%)	696 (8%)	2401 (36%)	2236 (37%)	8077 (13%)
**ESRD**	No	7072 (53%)	3046 (27%)	1575 (30%)	4603 (43%)	3448 (38%)	2067 (31%)	1723 (28%)	23534 (38%)
	Yes	14 (0%)	7 (0%)	8 (0%)	2 (0%)	0 (0%)	0 (0%)	2 (0%)	33 (0%)
	Missing	6163 (47%)	8269 (73%)	3678 (70%)	6068 (57%)	5672 (62%)	4685 (69%)	4398 (72%)	38933 (62%)
**Cardiovascular disease**	No	11347 (86%)	10555 (93%)	3196 (61%)	8927 (84%)	7267 (80%)	3751 (56%)	3448 (56%)	48491 (78%)
	Yes	44 (0%)	19 (0%)	24 (0%)	21 (0%)	14 (0%)	18 (0%)	6 (0%)	146 (0%)
	Missing	1858 (14%)	748 (7%)	2041 (39%)	1725 (16%)	1839 (20%)	2983 (44%)	2669 (44%)	13863 (22%)
**Smoker**	No	3877 (29%)	3348 (30%)	1061 (20%)	3105 (29%)	2107 (23%)	1413 (21%)	1164 (19%)	16075 (26%)
	Yes	2734 (21%)	1691 (15%)	682 (13%)	2301 (22%)	1628 (18%)	1050 (16%)	664 (11%)	10750 (17%)
	Missing	6638 (50%)	6283 (55%)	3518 (67%)	5267 (49%)	5385 (59%)	4289 (64%)	4295 (70%)	35675 (57%)
**Ethnicity**	White	8929 (67%)	6667 (59%)	3182 (60%)	6678 (63%)	6118 (67%)	3953 (59%)	3297 (54%)	38824 (62%)
	Black	2159 (16%)	3210 (28%)	1088 (21%)	1941 (18%)	1590 (17%)	1528 (23%)	1727 (28%)	13243 (21%)
	Hispanic	818 (6%)	452 (4%)	176 (3%)	680 (6%)	354 (4%)	298 (4%)	178 (3%)	2956 (5%)
	Asian	654 (5%)	561 (5%)	244 (5%)	576 (5%)	384 (4%)	364 (5%)	280 (5%)	3063 (5%)
	Other	111 (1%)	77 (1%)	170 (3%)	70 (1%)	92 (1%)	183 (3%)	180 (3%)	883 (1%)
	Unknown	578 (4%)	355 (3%)	401 (8%)	728 (7%)	582 (6%)	426 (6%)	461 (8%)	3531 (6%)
**Total**		**13249 (21%)**	**11322 (18%)**	**5261 (8%)**	**10673 (17%)**	**9120 (15%)**	**6752 (11%)**	**6123 (10%)**	**62500 (100%)**

Dolutegravir (DTG), Rilpivirine (RPV), Darunavir (DRV), Raltegravir (RAL), Elvitegravir (EVG), Efavirenz (EFV). ADM: AIDS defining malignancy. TB: Tuberculosis. NADM: Non-AIDS defining malignancy. HBsAG: Hepatitis B surface antigen. HCV: Hepatitis C virus. ESRD: End stage renal disease.

Supplementary table 2: Antiretroviral therapy (ART) regimens and backbones by ART start year

Variable	2013	2014	2015	2016	2017	2018	Total
**Third ART drug in regimen**							
Dolutegravir	27 (0%)	595 (5%)	3254 (26%)	3787 (36%)	3677 (41%)	1909 (40%)	13249 (21%)
Darunavir	3127 (24%)	2859 (22%)	1854 (15%)	1436 (14%)	1167 (13%)	879 (18%)	11322 (18%)
Raltegravir	1064 (8%)	1243 (10%)	1048 (9%)	874 (8%)	723 (8%)	309 (6%)	5261 (8%)
Elvitegravir	518 (4%)	1969 (15%)	2551 (21%)	2374 (23%)	2175 (24%)	1086 (23%)	10673 (17%)
Rilpivirine	2609 (20%)	2713 (21%)	1666 (14%)	998 (10%)	639 (7%)	495 (10%)	9120 (15%)
Efavirenz	3264 (25%)	1825 (14%)	992 (8%)	425 (4%)	194 (2%)	52 (1%)	6752 (11%)
Others	2598 (20%)	1570 (12%)	939 (8%)	603 (6%)	372 (4%)	41 (1%)	6123 (10%)
**Other drugs in regimen**							
FTC/TDF	10829 (82%)	10381 (81%)	8715 (71%)	6174 (59%)	3866 (43%)	2006 (42%)	41971 (67%)
3TC/ABC	1296 (10%)	1481 (12%)	2790 (23%)	2913 (28%)	2427 (27%)	944 (20%)	11851 (19%)
FTC/TAF	489 (4%)	406 (3%)	305 (2%)	1024 (10%)	2448 (27%)	1779 (37%)	6451 (10%)
Other	593 (4%)	506 (4%)	494 (4%)	386 (4%)	206 (2%)	42 (1%)	2227 (4%)
**Total**	**13207 (100%)**	**12774 (100%)**	**12304 (100%)**	**10497 (100%)**	**8947 (100%)**	**4771 (100%)**	**62500 (100%)**

Emtricitabine and Tenofovir disproxil (FTC/TDF), Lamivudine and Abacavir (3TC/ABC), Emtricitabine and Tenofovir alafenamide (FTC/TAF), and others.

Supplementary figure 1: Kaplan-Meier estimates of the cumulative incidence of loss-to-follow-up in the first four years of antiretroviral therapy, stratified by regimen (Reg).

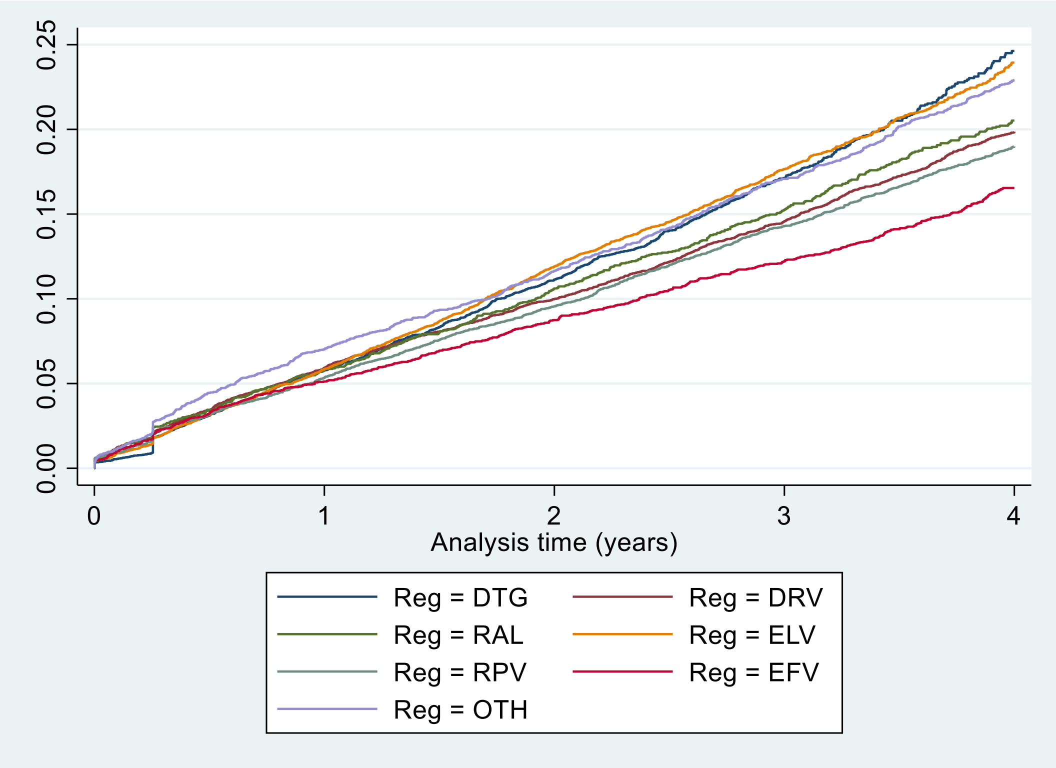

Dolutegravir (DTG), Darunavir (DRV), Raltegravir (RAL), Elvitegravir (EVG), Rilpivirine (RPV), Efavirenz (EFV), Other (OTH).

Supplementary table 3: Adjusted hazard ratios and 95% confidence intervals for each 3rd drug comparison, using dummy variables where there is missing data.

Hazard ratios (95% confidence intervals)								
**Analysis:**	**A) Crude**	**B) Adjusted**	**C) Adjusted**	**D) Adjusted**	**E) Meta-analysed across time periods**	**F1) Adjusted (2013-2015)**	**F2) Adjusted (2016-2018)**	**P-value 2013-2015 vs 2016-2018**
**Cohorts:**	**All**		**Those providing additional variables**			**Those providing additional variables**		
**Variables:**	**None**	**Main**	**Main**	**Additional**	**Additional**	**Additional**	**Additional**	
**Comparison**	**N=62,500**	**N=62,500**	**N=50,722**	**N=50,722**	**N=50,722**	**N=29,989**	**N=20,733**	
RPV vs DTG	0.45 (0.34-0.58)	0.77 (0.57-1.03)	0.74 (0.55-1.01)	0.81 (0.60-1.11)	0.76 (0.54-1.06)	0.79 (0.55-1.15)	0.59 (0.25-1.42)	0.544
DRV vs DTG	1.19 (0.97-1.47)	0.96 (0.77-1.20)	0.92 (0.73-1.16)	0.94 (0.74-1.18)	0.96 (0.75-1.22)	0.90 (0.66-1.22)	1.06 (0.72-1.57)	0.518
RAL vs DTG	2.32 (1.87-2.88)	1.79 (1.43-2.26)	1.73 (1.36-2.21)	1.58 (1.23-2.01)	1.49 (1.15-1.93)	1.58 (1.15-2.17)	1.33 (0.86-2.08)	0.535
ELV vs DTG	0.57 (0.45-0.72)	0.81 (0.63-1.03)	0.74 (0.57-0.97)	0.83 (0.64-1.08)	0.77 (0.58-1.02)	0.85 (0.60-1.21)	0.64 (0.40-1.02)	0.342
EFV vs DTG	0.62 (0.48-0.81)	0.69 (0.53-0.91)	0.71 (0.52-0.95)	0.75 (0.56-1.01)	0.71 (0.50-1.01)	0.74 (0.52-1.06)	0.18 (0.02-1.40)	0.198
RPV vs EVG	0.78 (0.60-1.03)	0.95 (0.72-1.26)	1.00 (0.74-1.35)	0.98 (0.73-1.32)	0.93 (0.68-1.27)	0.93 (0.67-1.30)	0.93 (0.38-2.30)	1.000
DRV vs EVG	2.09 (1.67-1.60)	1.19 (0.95-1.50)	1.23 (0.97-1.57)	1.13 (0.89-1.43)	1.19 (0.92-1.53)	1.06 (0.79-1.41)	1.66 (1.01-2.74)	0.128
RAL vs EVG	4.06 (3.23-5.09)	2.23 (1.76-2.82)	2.33 (1.82-2.99)	1.90 (1.48-2.45)	1.91 (1.47-2.48)	1.86 (1.38-2.51)	2.09 (1.21-3.62)	0.714
EFV vs EVG	1.09 (0.84-1.42)	0.86 (0.66-1.12)	0.95 (0.71-1.27)	0.91 (0.68-1.21)	0.86 (0.63-1.17)	0.88 (0.64-1.20)	0.29 (0.04-2.25)	0.286
DRV vs RPV	1.91 (1.53-2.39)	1.25 (0.98-1.61)	1.23 (0.95-1.61)	1.15 (0.88-1.51)	1.18 (0.90-1.55)	1.13 (0.85-1.51)	1.79 (0.74-4.30)	0.330
RAL vs RPV	5.17 (4.06-6.58)	2.34 (1.81-3.03)	2.33 (1.76-3.09)	1.94 (1.46-2.57)	2.02 (1.51-2.69)	1.99 (1.47-2.70)	2.25 (0.90-5.59)	0.803
EFV vs RPV	1.39 (1.06-1.83)	0.90 (0.68-1.20)	0.95 (0.70-1.29)	0.93 (0.68-1.26)	0.92 (0.67-1.26)	0.94 (0.68-1.29)	0.31 (0.04-2.73)	0.309
RAL vs DRV	1.95 (1.62-2.33)	1.87 (1.55-2.24)	1.89 (1.55-2.30)	1.68 (1.38-2.05)	1.66 (1.36-2.02)	1.76 (1.42-2.19)	1.26 (0.79-2.01)	0.203
EFV vs DRV	0.52 (0.42-0.65)	0.72 (0.57-0.90)	0.77 (0.60-0.99)	0.80 (0.63-1.03)	0.81 (0.62-1.06)	0.83 (0.63-1.08)	0.17 (0.02-1.33)	0.142
RAL vs EFV	3.72 (2.98-4.65)	2.59 (2.05-3.29)	2.45 (1.88-3.20)	2.09 (1.61-2.73)	2.18 (1.64-2.89)	2.13 (1.60-2.83)	7.24 (0.94-55.78)	0.245

Dolutegravir (DTG), Rilpivirine (RPV), Darunavir (DRV), Raltegravir (RAL), Elvitegravir (EVG), Efavirenz (EFV).

Analyses: A) Unadjusted models. B) Adjusted for the “main” variables, including all cohorts. C) Adjusted for the “main” variables, restricted to cohorts providing additional variables. D) Adjusted for “main” and “additional” variables, restricted to cohorts providing additional variables. E) Inverse-variance weighted meta-analyses of adjusted hazard ratios for 2013-15 (F1) and 2016-18 (F2).

Supplementary table 4: Adjusted odds ratios and 95% confidence intervals for each 3rd drug comparison, using dummy variables where there is missing data, and weighting by the inverse probability of loss-to-follow-up.

Comparison	Crude	Adjusted for main and additional variables
RPV vs DTG	0.44 (0.34-0.56)	0.94 (0.67-1.31)
DRV vs DTG	1.05 (0.87-1.28)	1.06 (0.81-1.39)
RAL vs DTG	2.52 (2.05-3.11)	1.83 (1.37-2.43)
EVG vs DTG	0.64 (0.51-0.81)	1.00 (0.74-1.34)
EFV vs DTG	1.01 (0.79-1.29)	0.91 (0.65-1.27)
RPV vs EVG	0.68 (0.51-0.90)	0.94 (0.69-1.29)
DRV vs EVG	1.64 (1.31-2.05)	1.07 (0.82-1.39)
RAL vs EVG	3.92 (3.08-4.98)	1.83 (1.38-2.43)
EFV vs EVG	1.56 (1.19-2.05)	0.91 (0.66-1.26)
DRV vs RPV	2.42 (1.77-2.98)	1.13 (0.85-1.51)
RAL vs RPV	5.80 (4.45-7.56)	1.95 (1.43-2.66)
EFV vs RPV	2.31 (1.72-3.11)	0.97 (0.70-1.35)
RAL vs DRV	2.40 (1.97-1.15)	1.72 (1.35-2.17)
EFV vs DRV	0.95 (0.76-1.20)	0.85 (0.65-1.12)
RAL vs EFV	2.51 (1.96-3.22)	2.01 (1.50-2.70)

Dolutegravir (DTG), Rilpivirine (RPV), Darunavir (DRV), Raltegravir (RAL), Elvitegravir (EVG), Efavirenz (EFV).

*Analyses: D) Adjusted for main and additional variables, restricted to cohorts providing additional variables.

Supplementary table 5: Hazard ratios (95% CI) comparing mortality after starting ART between each 3rd drug, separately for PWH who did and did not present late for treatment (defined as CD4≥350 cells/mm^3 and no prior AIDS and viral load<100,000 copies/mL).

	Not presenting late (N=26,032)		Presenting late (N=24,690)		P-value comparing adjusted estimates
**Comparison**	**Crude**	**Adjusted**	**Crude**	**Adjusted**	
RPV vs DTG	0.63 (0.37-1.07)	0.86 (0.46-1.60)	0.51 (0.36-0.72)	0.98 (0.68-1.42)	0.724
DRV vs DTG	0.85 (0.49-1.47)	1.01 (0.56-1.84)	0.96 (0.75-1.21)	0.96 (0.74-1.23)	0.878
RAL vs DTG	2.77 (1.62-4.73)	2.77 (1.54-5.01)	1.87 (1.45-2.40)	1.49 (1.14-1.94)	0.060
EVG vs DTG	0.67 (0.40-1.10)	0.97 (0.54-1.75)	0.53 (0.40-0.70)	0.83 (0.61-1.12)	0.644
EFV vs DTG	0.79 (0.44-1.40)	0.94 (0.48-1.83)	0.57 (0.41-0.78)	0.75 (0.54-1.06)	0.555
RPV vs EVG	0.94 (0.56-1.58)	0.88 (0.52-1.50)	0.97 (0.68-1.39)	1.18 (0.82-1.72)	0.374
DRV vs EVG	1.27 (0.74-2.17)	1.04 (0.60-1.81)	1.80 (1.38-2.35)	1.15 (0.88-1.51)	0.748
RAL vs EVG	4.15 (2.47-6.96)	2.85 (1.67-4.87)	3.52 (2.67-4.64)	1.79 (1.34-2.39)	0.134
EFV vs EVG	1.18 (0.68-2.04)	0.97 (0.54-1.72)	1.07 (0.77-1.49)	0.91 (0.65-1.28)	0.852
DRV vs RPV	1.35 (0.81-2.25)	1.18 (0.69-2.02)	1.86 (1.37-2.52)	0.97 (0.70-1.35)	0.542
RAL vs RPV	4.42 (2.70-7.24)	3.24 (1.91-5.48)	3.63 (2.64-4.99)	1.51 (1.07-2.13)	0.017
EFV vs RPV	1.25 (0.74-2.12)	1.09 (0.63-1.90)	1.11 (0.77-1.59)	0.77 (0.53-1.12)	0.307
RAL vs DRV	3.27 (1.96-5.47)	2.74 (1.62-4.64)	1.95 (1.59-2.39)	1.55 (1.25-1.93)	0.050
EFV vs DRV	0.93 (0.53-1.62)	0.93 (0.52-1.66)	0.59 (0.45-0.78)	0.79 (0.59-1.05)	0.622
RAL vs EFV	3.52 (2.11-5.87)	2.96 (1.67-5.22)	3.29 (2.48-4.35)	1.97 (1.46-2.67)	0.216

Dolutegravir (DTG), Rilpivirine (RPV), Darunavir (DRV), Raltegravir (RAL), Elvitegravir (EVG), Efavirenz (EFV).

*Analyses: D) Adjusted for main and additional variables, restricted to cohorts providing both main and additional variables.

Supplementary table 6: Hazard ratios (95% CIs) comparing time to viral suppression after starting ART between each 3rd drug, among 50,722 PWH included in cohorts that provided main and additional variables.

Comparison	Crude	Adjusted for main and additional variables
RPV vs DTG	0.84 (0.59-0.62)	0.61 (0.59-0.63)
DRV vs DTG	0.61 (0.59-0.62)	0.60 (0.58-0.62)
RAL vs DTG	0.71 (0.67-0.74)	0.78 (0.74-0.82)
EVG vs DTG	0.93 (0.90-0.96)	0.88 (0.85-0.92)
EFV vs DTG	0.65 (0.63-0.68)	0.64 (0.61-0.67)
RPV vs EVG	0.91 (0.88-0.94)	0.69 (0.66-0.72)
DRV vs EVG	0.65 (0.63-0.67)	0.68 (0.65-0.70)
RAL vs EVG	0.76 (0.73-0.80)	0.89 (0.84-0.93)
EFV vs EVG	0.70 (0.68-0.73)	0.72 (0.69-0.75)
DRV vs RPV	0.72 (0.70-0.74)	0.98 (0.95-1.01)
RAL vs RPV	0.84 (0.80-0.88)	1.28 (1.22-1.35)
EFV vs RPV	0.77 (0.75-0.80)	1.05 (1.01-1.09)
RAL vs DRV	1.17 (1.12-1.22)	1.31 (1.25-1.37)
EFV vs DRV	1.53 (1.49-1.58)	1.07 (1.03-1.11)
RAL vs EFV	1.08 (1.03-1.14)	1.22 (1.16-1.29)

Dolutegravir (DTG), Rilpivirine (RPV), Darunavir (DRV), Raltegravir (RAL), Elvitegravir (EVG), Efavirenz (EFV).

*Analyses: D) Adjusted for main and additional variables, restricted to cohorts providing additional variables.

Supplementary table 7: Hazard ratios (95% CIs) comparing time to viral failure after starting ART between each 3rd drug, among 50,722 PWH included in cohorts that provided main and additional variables.

**Comparison**	**Crude**	**Adjusted for main and additional variables**
RPV vs DTG	1.09 (0.99-1.22)	1.31 (1.16-1.48)
DRV vs DTG	1.69 (1.55-1.85)	1.50 (1.35-1.66)
RAL vs DTG	1.92 (1.71-2.16)	1.60 (1.41-1.81)
EVG vs DTG	1.30 (1.17-1.44)	1.39 (1.23-1.56)
EFV vs DTG	1.76 (1.58-1.96)	1.56 (1.38-1.75)
RPV vs EVG	0.85 (0.77-0.94)	0.94 (0.85-1.05)
DRV vs EVG	1.30 (1.20-1.42)	1.08 (0.99-1.19)
RAL vs EVG	1.48 (1.32-1.66)	1.15 (1.02-1.30)
EFV vs EVG	1.36 (1.23-1.51)	1.12 (1.01-1.25)
DRV vs RPV	1.54 (1.41-1.69)	1.15 (1.04-1.27)
RAL vs RPV	1.75 (1.56-1.96)	1.22 (1.08-1.38)
EFV vs RPV	1.61 (1.44-1.79)	1.19 (1.07-1.33)
RAL vs DRV	1.14 (1.03-1.26)	1.06 (0.96-1.18)
EFV vs DRV	1.04 (0.95-1.14)	1.04 (0.94-1.14)
RAL vs EFV	1.09 (0.97-1.23)	1.03 (0.91-1.15)

Dolutegravir (DTG), Rilpivirine (RPV), Darunavir (DRV), Raltegravir (RAL), Elvitegravir (EVG), Efavirenz (EFV).

*Analyses: D) Adjusted for main and additional variables, restricted to cohorts providing additional variables.
